# Supplementary material for: Fallopian Tube Basal Stem Cells Reproducing the Epithelial Sheets In Vitro—Stem Cell of Fallopian Epithelium
Source: Biomolecules. 2020 Sep 3;10(9):1270. doi: 10.3390/biom10091270 (PMC7565394; doi:10.3390/biom10091270)
Supplement: Supplementary file 1 [file biomolecules-10-01270-s001.zip › supplementary figures.pdf]

Supplementary figure 1 Identification of primary FTECs.

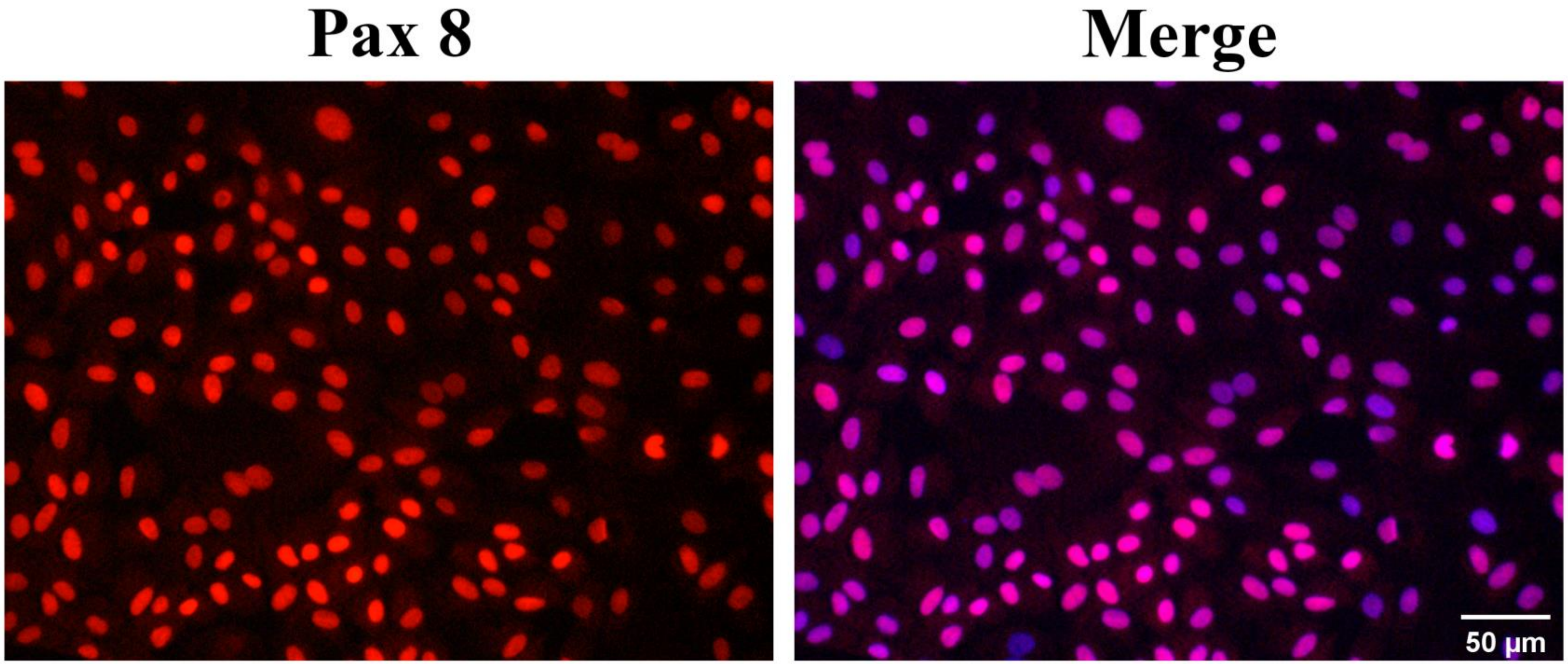

Primary FTECs were stained with Pax8, and DAPI Scale bar: 50 µm.

Supplementary figure 2 Purification of FTECs co-cultured with MEFs

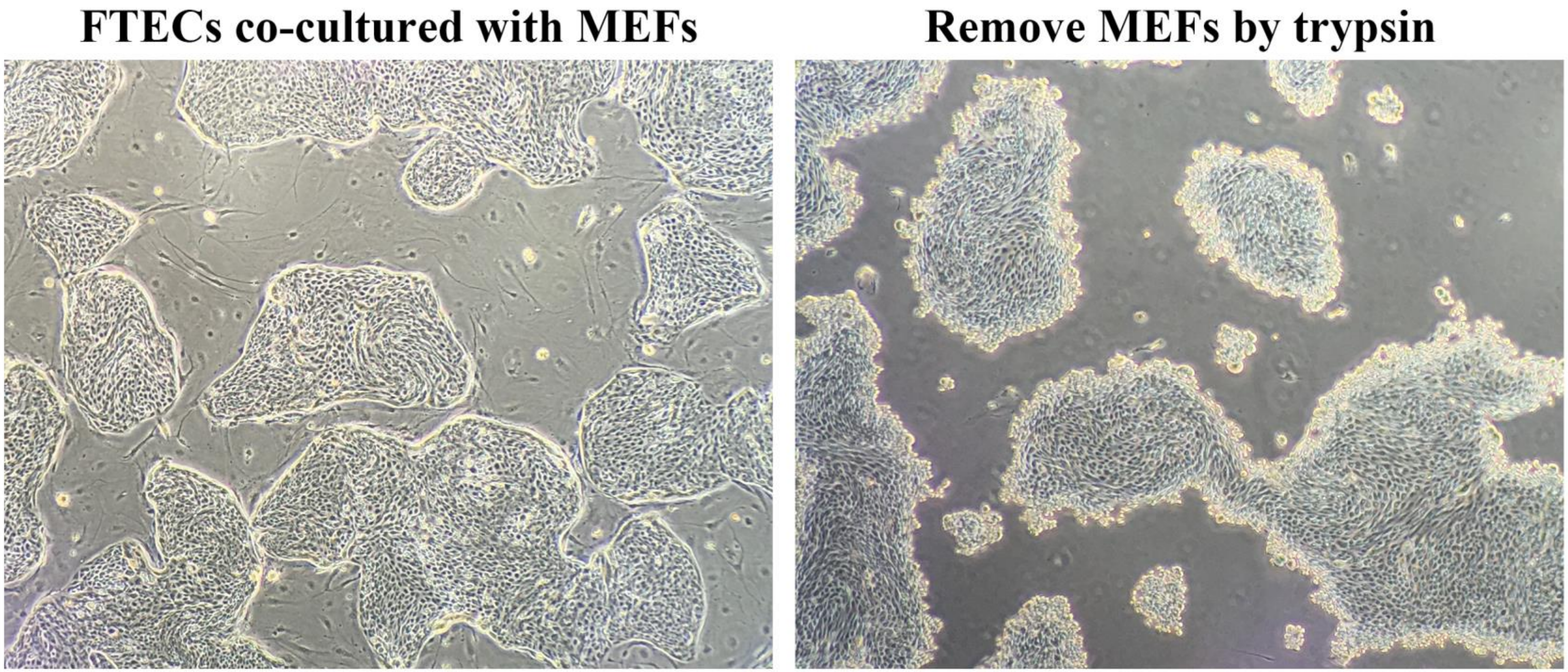

FTECs were co-cultured with MEFs for long-term expansion and MEFs were removed by differential trypsinization.
